# Supplementary material for: Chromothripsis during telomere crisis is independent of NHEJ, and consistent with a replicative origin
Source: Genome Res. 2019 May;29(5):737–49. doi: 10.1101/gr.240705.118 (PMC6499312; doi:10.1101/gr.240705.118)
Supplement: Supplemental Material [file supp_gr.240705.118_Supplemental_file_1.zip › contigs/annotated_contigs/DB67/contig.4.DB67_length_273_mean_cov_2.35531135531.docx]

**DB67_length_273_mean_cov_2.35531135531**

```
GCGACGCAAGTCGCCGTACGCCAAGAATGCGCCAACACACCCCCGCCGGGGTA|AAAGAGCGAGCCTCTGTAGCAAAAAAAAAAAA|GC
                                                      >chr1:37776441-37776473 - E=7e-02
CA|GGCATGGTGACAGGCACCTGTAACCCCAGCTACTCAGGAGGCTGAGGCAGGAGAATTGCTTGAGCCTGGGAGGCGGAGGTTGCAGT
   >chr6:141912376-141912478 - E=5e-32
GAGCCGAGATCTTGCT|AGG|GCACTCGAAGCGGGGGGACAGAGCGAGACTCC|GGCGCAGAAAACGAAGGAGG|GCGCAGGCGTGGTG
                    >chr3:49126516-49126548 - E=7e-02                   >chr2:4312393
GCTCACGCCTATA3-43123960 -
```
